# Expectations and Prospects of Young Adult Caregivers Regarding the Support of Professionals: A Qualitative Focus Group Study

**DOI:** 10.3390/ijerph17124299

**Published:** 2020-06-16

**Authors:** Hinke M. van der Werf, Wolter Paans, Geertjan Emmens, Anneke L. Francke, Petrie F. Roodbol, Marie Louise A. Luttik

**Affiliations:** 1 Research Group Nursing Diagnostics, Hanze University of Applied Sciences, 9714 CA Groningen, The Netherlands; w.paans@pl.hanze.nl (W.P.); g.emmens@pl.hanze.nl (G.E.); m.l.a.luttik@pl.hanze.nl (M.L.A.L.); 2 Nivel, Netherlands Institute for Health Services Research, 3500 BN Utrecht, The Netherlands; A.Francke@nivel.nl; 3 APH Amsterdam Public Health Research Institute/Vrije Universiteit Amsterdam, 1007 MB Amsterdam, The Netherlands; 4 Faculty of Medical Sciences, University Medical Center Groningen, 9700 RB Groningen, The Netherlands; petrieroodbol@gmail.com

**Keywords:** young adults, caregiving, adulthood, family care, support, professionals

## Abstract

There is a lack of service provision for young adult caregivers (18–25 years of age). This study aims to describe the expectations and prospects of young adult caregivers regarding support from health and education professionals. A qualitative focus group design was used. Twenty-five young Dutch adults (aged 18–25 years) who were growing up with a chronically ill family member participated in one of seven focus groups. Qualitative inductive analysis was used to identify codes and main themes. Two overarching themes with five sub-themes emerged from the focus group discussions. The overarching themes are: the ‘process of approaching young adults’ and the ‘types of support these young adults require’. The process of approaching young adults contains the sub-themes: ‘recognition, attention, and listening’, ‘open-minded attitude’, ‘reliability’, and ‘respecting autonomy’. The types of support this group requires contains the sub-themes: ‘information and emotional support’. Health and education professionals should first and foremost be aware and listen to young adult caregivers, pay attention to them, have an open-minded attitude, respect their autonomy, and have the knowledge to provide them with information and emotional support. Further research could yield comprehensive insights into how professionals can meet these requirements and whether these results apply to male young adult caregivers and young adult caregivers not enrolled in a healthcare-related study program.

## 1. Introduction

Growing up with a chronically ill family member can have a negative impact in terms of mental health problems [1], stress, problems in the parent–child relationship, developmental problems, and poorer school results [2,3]. Young adults up to the age of 25 growing up with a chronically ill family member are an under-identified age bracket that faces challenges [4]. Their vulnerability is twofold, defined by both their life stage and their family situation. The life stage of young adults is characterized by change and the exploration and development of their own identity [5]. The presence of supportive and well-functioning families is particularly important in this developmental stage [5,6]. Families in which a family member has a chronic illness may be unable to offer emotional or financial support.

As a result, young adults growing up in this environment may require additional attention, for example from healthcare and school professionals such as general practitioners, community nurses, social workers, or school psychologists. Unfortunately, various studies reveal that these young adults are overlooked by professionals despite their wish for assistance or information on their family member’s illness [3,7,8,9]. Furthermore, when support is available for young caregivers, it is not always considered suitable. This is mainly attributed to miscommunication between young caregivers and professionals [4,7,9,10]. A lack of understanding and the feeling of being judged for their family member’s illness or care tasks that are being performed can lead young caregivers to avoid or cancel follow-up appointments [9].

Studies among professionals describe the lack of sufficient knowledge about how to support this specific group of caregivers [11,12]. In these studies, professionals reported a need for skilled outreach workers to work closely with young caregivers and a need for more interdisciplinary and inter-professional collaboration. They also mentioned a lack of adequate screening tools and age-based information materials. 

The need for age-based support and information materials was also found in a study by Becker and Becker [4]. These authors described the lack of adequate service provision for young adult carers aged 18–25 years; this specific age group felt too old to attend social events for younger carers and too young for adult carer events. The support needs of young adults are of a different nature because of their life stage, which is more focused on developing their own identity [5].

The aim of this study is to investigate the expectations and prospects of young adult caregivers regarding support from professionals to manage their own health and wellbeing. Since there is no clear designation of which professionals are responsible or competent to support young caregivers [12], we have broadly defined professionals in both health and educational settings as general practitioners, community nurses, social workers, psychologists, and teachers. All these professionals may encounter young adult carers. 

### Research Question

What ‘expectations and prospects’ do young adult caregivers have regarding support from professionals?

## 2. Materials and Methods

### 2.1. Design

We used a qualitative descriptive focus group design. We chose focus groups as the data collection method, since comments from others can trigger memories and reflections in participants and therefore also generate more information than in-depth individual interviews [13].

### 2.2. Participant Recruitment

The focus in this study is on young adult caregivers enrolled in a Bachelor’s program or secondary vocational education. We approached this specific subgroup of young adult caregivers for practical reasons because their enrolment in an educational program made them more reachable for recruitment. The participants were recruited from an online survey sent out in September 2017 to all students (*n* = 5997) registered at a university of applied sciences or a school for secondary vocational education in the northern Netherlands [14]. Schools for secondary vocational education have a practical approach to learning that is similar to level 4 of the International Standard Classification of Education, while the universities of applied sciences offer a Bachelor’s degree similar to level 6 of the International Standard Classification of Education [15]. 

Young adults growing up with a chronically ill family member were asked if they were willing to participate in a focus group. Those who agreed were asked to share their email address. In total, 40 eligible participants were identified and deemed eligible to take part in a focus group. Over the next two weeks, participation information and invitations to schedule an appointment were sent to them by email. 

### 2.3. Data Collection

Due to the sensitive nature of the topic and the age of the participants, we chose to organize small focus groups with a maximum of five participants [16,17,18]. All focus groups were held in a separate, quiet room at the university or school where the participants were studying. The focus group sessions took 90 to 120 min and were audiotaped. 

We used a semi-structured topic list, based on several studies [4,19,20] and research outcomes published in earlier studies [14] to cover the main topics relating to the aim of the study. A draft topic list was discussed with experts in the area of education and youth care, nurses (*n* = 8), lecturers (*n* = 5), and psychologists (*n* = 2), in order to judge the relevance of the topics and to evaluate the possible emotional impact on participants. After this feedback, the draft topic list was pilot-tested for comprehensibility and feasibility by six young adult caregivers. The content was discussed and adjusted in response to their feedback. 

The focus groups were led by two of the authors of this paper (G.E. and H.M.W.), who are experienced in leading dynamic group discussions with young adults. During the focus groups, the focus group leaders aimed to create a good atmosphere and to establish trust. A funnel approach was used to give participants time to feel comfortable about contributing to the discussion. The focus group opened with broad questions such as ‘Can you introduce yourself and tell us why you decided to take part in this focus group?’ and then moved on to more specific questions about the needs of young adult caregivers. We divided the topic needs into the following questions:

- Do/did you need support?
- Did you receive support?
- If yes, can you tell something about the support and the effect of the support?
- What are your expectations regarding professionals?
- What advice do you want to give to professionals who deal with young adult caregivers?

Each focus group session was followed by an oral evaluation with each individual student, since participation in a focus group can evoke unprocessed emotions, re-experiences, and stress. Referrals for further support were made where necessary and with the student’s consent (six participants).

### 2.4. Data Analysis

The first author (H.M.W.) transcribed all focus group sessions verbatim and anonymized names and other identifying participant information prior to analysis. The themes that emerged in the transcripts of the focus groups were discussed by H.M.W., G.E., and M.L.A.L. immediately after each focus group session and further explored in subsequent sessions until data saturation was achieved. Qualitative inductive content analysis was carried out in line with the steps described by Hennink et al. [21]. Before taking the first step, H.M.W. and M.L.A.L. read the transcripts several times in order to familiarize themselves with the data. In step one of the data analysis, sections of texts addressing expectations and prospects regarding professionals were identified and coded by H.M.W. and M.L.A.L. In step two, codes were individually and critically examined, and overlapping codes were further refined and grouped together by the aforementioned researchers. The coding process was supported by the ATLAS.ti 8 (8.3.16) software program (ATLAS.ti Scientific Software Development GmbH, Berlin, Germany). Following this inductive coding step, H.M.W., M.L.A.L., W.P., and P.F.R. sorted subcodes with similar characteristics into meaningful overarching themes and compared them in step three. These themes were discussed within the author group for accurate naming, resulting in the themes of ‘recognition, attention, and listening’, ‘open-minded attitude’, ‘reliability’, ‘respecting autonomy’, and ‘information and emotional support’.

### 2.5. Ethical Considerations

Prior to participation, the participants received written and verbal information on the aim and procedures of the focus groups. Written informed consent was obtained before the start of each focus group session. Participation in this study was voluntary, and the participants were told that they could withdraw from further participation or choose not to answer certain questions without giving a reason. The study was approved by the Ethical Review Board of the Dutch Association for Medical Education (NVMO) (#940).

## 3. Results

### 3.1. General Findings

Twenty-five of the 40 eligible young adults agreed to take part in a focus group session between November 2017 and November 2018. Descriptive characteristics of the participants are summarized in Table 1. Reasons for non-participation after showing initial interest related to practical or logistical issues (e.g., prohibitive internship or work schedules).

Most of the 25 participating students were female nursing students with family members suffering from both mental and physical disorders such as cancer, cardiovascular disease, depression, and addiction. All participants had experience with professional support and could state whether or not this support was adequate. The participants mentioned the attitude of the professional as important in order to approach them, start a conversation, and ultimately to be able to accept their support. Therefore, the themes were divided into the ‘process of approaching young adult caregivers’ and the ‘types of support these young adult caregivers require’.

### 3.2. Process of Approaching Young Adult Caregivers

Regarding the approach to young adults, all participants mentioned the importance of professionals paying attention to their attitude. Although participants mentioned various experiences with such professionals as general practitioners, community nurses, social workers, school psychologists, and teachers, they often struggled with the attitude of these professionals. As a result, they did not feel compelled to continue their request for support, and most of them avoided further contact. Conversely, an appropriate attitude led to openness to tell their story, as stated by one participant, who needed a trusting relationship with a professional before she could tell her story:

And I actually need to have a connection with a care professional. We need to click, I think. If I click with them, I can tell them anything. But if I don’t click, then, um, I just rush through things a bit.

*(participant 13, FG4)*

Important themes that emerged regarding the approach to young adults were recognition, attention and listening, an open-minded attitude, reliability, and respecting autonomy.

#### 3.2.1. Recognition, Attention, and Listening

Recognition, attention, and listening at the start of contact invite young adults growing up with a chronically ill family member to talk openly about their family situation. Recognition can be defined as identifying young adults growing up with a chronically ill family member and having a family-focused approach. Participants felt that professionals did not acknowledge them, talked predominantly with and about their chronically ill family member, and paid no attention to them, as stated by one participant:

I think that that’s also very important, to keep asking: ‘How are you?’ And: ‘What can we do for you?’ And less about: ‘What can we do for your father or mother?’

*(participant 3, FG1)*

Attention is defined as a genuine interest in the family situation and the consequences for young adults growing up with a chronically ill family member. Attention is necessary to invite them to talk openly about their family situation and to understand the needs of these young adult caregivers, as mentioned by one participant:

So, if you ask some questions that show that you’re interested, then it often starts to happen. Then each time there’s a little bit more.

*(participant 12, FG4)*

All participants also mentioned listening as an important aspect of the approach; it can be seen as an expression of sincere empathy. It is conspicuous that all participants experienced situations in which they felt that professionals were not listening, although listening was mentioned as one of the most important interventions, as stated by one participant:

Just listening, offering a listening ear, I think that’s the main thing.

*(participant 1, FG1)*

#### 3.2.2. Open-Minded Attitude

Building a trusting relationship with these young adults seems to be a delicate process; contact can vanish just as quickly as it arises. The professional’s attitude was important for consolidating this contact and preventing resistance from emerging. An open-minded attitude is essential for these young adults to tell their story openly. It can be defined as an attitude that does not involve judgement or stereotyping. Participants reported feeling judged because of their burden and family situation, as stated by one participant:

No judgement about your story, you have to be very careful about that because of course we pick up on that very quickly.

*(participant 16, FG5)*

Stereotyping them as a vulnerable target group was also not conducive to maintaining contact, as stated by one participant:

The questions that were asked, I’ve always felt that I don’t want pity. I don’t need to be pitied. Definitely not pitied!

*(participant 20, FG6)*

#### 3.2.3. Reliability

Reliability is also essential for young adults to stay in contact. Reliability can be defined as complying with agreements and being able to handle conversations confidentially. Participants chose to avoid or cancel follow-up appointments if agreements were not complied with, as stated by one participant:

And what, um, and if I look at other professionals, like my GP who always does what he says, I would always go back. But the ‘WE team’ that we had then, they didn’t stick to their agreements at all, so I’m never going there again. Because they don’t do what they say.

*(participant 8, FG3)*

Resistance and the cancellation of follow-up appointments also occurred if professionals did not treat conversations confidentially, as stated by one participant:

Well, at school I once had a conversation with the confidential counsellor and they sent a CC to the teacher. Then you can forget about it as far as I’m concerned.

*(participant 25, FG7)*

#### 3.2.4. Respecting Autonomy

Despite their young age, all participants reported that they wanted to carry out care tasks and felt responsible for their chronically ill family member. They wanted respect for their choices, as most participants (21) described a strong wish to continue the care tasks for their chronically ill family member, as stated by one participant:

There’s also a very strong desire to keep helping.

*(participant 11, FG4)*

Respecting their autonomy can be defined as respecting their responsibility, their wish to continue their care tasks, and a reluctance to be given unsolicited advice or to resort to solutions too quickly. The wish to continue care tasks was often in conflict with the professionals’ wish to take over those tasks. Such an attitude resulted in misunderstanding and conflict, causing resistance and withdrawal from further contact. Participants described their responsibility for their family member and not being acknowledged in their care role. Not recognizing their care role leads to a feeling of misunderstanding, resulting in resistance to support, as stated by one participant:

You don’t want someone to take away the problem either, not in any way.

*(participant 13, FG4)*

Respecting their autonomy and their need to be taken seriously by professionals was mentioned by all participants. Most participants (23) had the opposite experience, resulting in resistance and misunderstanding, as indicated by one participant, who experienced unsolicited advice:

She came down from on high, as though she’d take care of it. And that doesn’t work with me at all, in fact, it had a counterproductive effect.

*(participant 22, FG7)*

Participants reported that professionals resorted to solutions too quickly because of their age and family situation. One participant described a misunderstanding with a professional who wanted to solve her problems immediately:

And by chance, that female psychologist who asked: ‘Why have you come here?’ Tell me. Go on. She really wanted to solve the problem on the spot.

*(participant 17, FG5)*

### 3.3. Types of Support Required

The first step is the actual request for support when professionals conform to the approach of recognition, attention, and listening, an open-minded attitude, reliability, and respect for autonomy. The second step is receiving eventual concrete instrumental support such as information and emotional support.

#### Information and Emotional Support

The participants mentioned requirements regarding information and emotional support from general practitioners, community nurses, social workers, school psychologists, and teachers to support them in their family situation. Most participants reported that their family situation was hard to understand for professionals. Some said that it was important to have information about family roles and family dynamics if there was an ill person in the family in order to explain the family behavior, as stated by one participant:

I think that as a care professional it’s important to realize that you have these roles in such families and that every person operates differently in the family. I think it’s important to bring this knowledge with you so that you can also understand the carer.

*(participant 4, FG2)*

Participants appreciated the provision of information about illnesses and their consequences and symptoms. In particular, participants growing up with a chronically ill family member suffering from a mental disorder had questions about their family member’s behavior and how they should respond. Most participants said that they had found the information themselves but would prefer to have received some information about their family member’s illness, as stated by one participant:

An explanation of how it all works. Because, of course, in the beginning it was all new and it was all very strange. You learn to deal with it, you learn what’s going on, but to have someone who says this is normal, this is what happens, that would have been very good.

*(participant 5, FG2)*

In addition to the information about family patterns and illnesses, some participants mentioned specific information regarding psychological support, for example coping strategies, as stated by one participant:

How you should deal with anxiety. Of course, they can’t tell you that, but that you also consider how you find it and the kind of things you’ve done and how you felt about it. And whether you’ll do the same thing again next time.

*(participant 22, FG6)*

Other participants said they needed support for ‘how to survive’. Their lives were so busy with caring that there was no time to think about the future. A professional could help them to create a vision for their future, as stated by one participant:

Yes, because sometimes you get stuck, after my brother’s accident, I just got stuck. And then I just needed some kind of vision of how I should go on. Some kind of rest, but also just okay, I can get through this. How can I move on, tips like that.

*(participant 7, FG3)*

## 4. Discussion

The findings of this study indicate that young adult caregivers often needed a different approach from what they usually received. All participants expressed a need for support from professionals and they were able to provide examples of good and bad practices. The main theme was the need for an appropriate approach by professionals when communicating with young adult caregivers.

The desired approach to young adult caregivers involves a number of conditions. First, recognition, attention, and listening were mentioned as being important in order to encourage young adults growing up with a chronically ill family member to talk openly about their family situation. A family-focused approach with attention paid to young adult carers is necessary for understanding their needs, according to Metzing-Blau and Schnepp [22]. This finding has also been established in various studies [3,7,8,9], which found that a lack of recognition and attention for this target group resulted in being overlooked by professionals, whereas these young carers longed for support. Second, participants mentioned feeling vulnerable when initiating conversations about their family situation, inside and outside a support context. This finding is consistent with the study by Eley [10], who found that young caregivers’ fear of having their family story seen by teachers was an excuse for not fulfilling requirements for school attendance/assignments. More broadly, participants felt an aversion to being judged or criticized for their family members’ illness or the care tasks that they performed, which is similar to the findings of Moore and McArthur [9]. This creates a barrier both to contacting professionals for support and to opening up while receiving support. An open-minded attitude on the part of professionals involving an awareness of this vulnerability is key to reducing the barriers to initial contact [12]. Third, participants mentioned the importance of reliability and a professional with a trustworthy attitude as a prerequisite for them to open up during support, as also found by Becker and Becker [4].

Lastly, participants deemed support insufficient when professionals jumped to solutions too quickly instead of engaging the young caregivers in the process as autonomous individuals. As a result, these young adults did not feel that they were being taken seriously and they therefore disengaged or refrained from further contact, a finding also found in the study by Moore and McArthur [9]. Arnett [5] explains this finding in terms of the developmental stage of emerging adulthood. In this life stage, young adults develop their own identity, aim to become independent of their parents, and start to bear their own responsibility. Young adult caregivers struggle with loosening the ties to their family, which might hinder the usual process of growing up. However, it can also be seen as a double-edged sword, since these young caregivers are often capable of carrying great responsibility [4,23,24] and generally behave more maturely than peers who have not grown up with a chronically ill family member [25,26]. The opposite also holds true: professionals who acknowledge both the participant’s strengths and needs were more successful at communicating with the participants in this study.

We identified four distinct topics where young caregivers experienced a knowledge gap. First, they wanted more information on the illness of their family member, a finding consistent with other research [7,12]. Second, they sought information about coping strategies for dealing with grief and fear caused by their family situation. Various studies suggest that a lack of adequate coping strategies may lead to problems of both an externalized (aggression and delinquency) and an internalized (anxiety, depression, and social withdrawal) nature [27,28,29]. Providing information about coping strategies might prevent these problems. Third, as mentioned above in relation to the study by Arnett [5], they needed advice on how to set out a long-term strategy for their future. Due to illness within their family, young adult caregivers may not be able to discuss their strategy for the future within their family, resulting in a day-by-day approach to living. Lastly, they requested information on normal family functioning, family dynamics, and roles to mirror and thus better understand what was happening in their own family situation. Discussing family functioning and roles within the families still seems to be a taboo. Making the illness a taboo topic or lack of communication could result in mental and family relationship problems [1,2]. Information on family patterns, family functioning, and roles can be a start for openly discussing family roles within the family, resulting in improved well-being and family functioning [22,30,31].

### 4.1. Clinical and Research Implications

Our findings suggest that young adult caregivers require specific attention in the development of care policies. Participants reported that most of the support focused on the well-being of their chronically ill family member and that support for other family members was only provided when explicitly requested. Most healthcare professionals still focus mainly on providing individual support to the ill family member and pay little attention to the family as a whole. The concept of family health conversations [31] could be a possible intervention in this context for healthcare professionals. Here, the professional discusses the care situation with the family as a whole, incorporating the experiences of and consequences for each family member. By taking on this contextual understanding, family health conversations could prevent the incomprehension felt by young caregivers [32,33].

The education professional can also play a role in identifying and supporting these young adult caregivers. Young adult caregivers do not always ask for help themselves, which makes it important to pay attention to this target group within the educational institution. Educational professionals are encouraged to initiate a conversation with the student when signalizing notable behaviors or poorer school results. This conversation needs to focus on the students’ wellbeing and their home situation [10]. We would recommend the development of specific guidelines for educational professionals on how to recognize and approach young adult caregivers.

In this study, we have broadly discussed the role of professionals. A topic for further research could be whether a specific group of professionals might be especially suited for this task. For example, homecare nurses who can easily contact a family to organize family health conversations or school nurses/counsellors who can support young adult caregivers enrolled in education in order to prevent delay in or withdrawal from their studies [4,12,34]. Interdisciplinary and inter-professional collaboration among professionals is necessary to finetune the required support. We recommend that future research should investigate the specific needs and wishes of these professionals regarding the provision of support and, subsequently, the availability of this support within the current healthcare and education systems.

### 4.2. Strengths and Limitations

The focus groups proved to be a suitable method for gaining insight into the prospects and expectations of young adult caregivers regarding professionals. Despite the personal and emotional stories, the participants stated that they were glad of the attention given to this topic. A remarkable openness during the sessions resulted in rich data.

The invisibility of young adult caregivers means that it is also difficult to recruit them for research studies. To facilitate recruitment, we focused on approaching students who were reachable through their educational institution. These results may not be applicable to young working adults who are also growing up with a chronically ill family member but who are no longer in education.

The majority of the respondents were female students (24), and most of these students (20) were enrolled in a healthcare-related study program which limits the generalizability of the results. The high number of participants enrolled in a healthcare-related study could be explained by the so-called ‘care identity’ described by Becker and Becker [4]. They found that young adult caregivers are more likely to be drawn towards care-related careers. The choice of their study could be so self-evident because of their care experiences and knowledge about illness that no other study options are considered. Further research should investigate whether these results also apply to young adult caregivers not enrolled in a healthcare- related study program.

The gender imbalance with the majority of female caregivers is known in other studies among older caregivers with the same majority of female caregivers [35,36]. These results are often explained by the traditional caregiving role and behavior of women. In the case of illness, women still seem to take on the (emotional) care tasks, while men take often care of the practical matters [37]. Perhaps this also led to the gender imbalance within this study, that male participants were less attracted to the definition of young adult caregiver, despite growing up with a chronically ill family member and managing all kinds of practical matters. It would be interesting to investigate whether female caregivers are more inclined to identify themselves as being a (young adult) caregiver or whether they feel more able, for example, to discuss their chronically ill family member.

Furthermore, 22 of the 25 participants have a Western European cultural background. The cultural background might influence the needs of young adult caregivers, but this was outside the scope of this study and can be seen as an important topic for further research.

## 5. Conclusions

In this study, young adults growing up with a chronically ill family member clearly indicated that professionals need to recognize young adult carers as a group requiring specific attention. They mentioned having an open-minded attitude, listening to them, and respecting their autonomy as important requirements in order for them to talk openly about their family situation. These findings suggest that young adult caregivers require specific attention in the development of education and care policies. Healthcare professionals should pay attention to the family as a whole, instead of providing individual support to the ill family member. Furthermore, specific guidelines for educational professionals on how to recognize, approach, and support young adult caregivers may be needed in order to support educational professionals. Future research should focus on whether professionals in the current healthcare system and educational institutions are able to meet the requirements that young adult carers defined in this study. Furthermore, it would be interesting to investigate whether these results apply to male young adult caregivers and young adult caregivers not enrolled in a healthcare-related study program.

## Figures and Tables

**Table 1:** Demographic data of the participants who attended a focus group.

Variables	Mean (Standard Deviation)
Age (years)	21.4 (1.7). *n*
Gender	
Female	24
Male	1
Level of Education	
University of applied sciences	14
Secondary vocational education	11
Study	
Non-healthcare-related studies (Law and Communication, Media, and ICT)	5
Healthcare-related studies (Nursing and Social Work)	20
Type of Family Member Being Ill	
Mother	5
Father	5
Sibling	4
Other ^†^	4
Multiple	7
Type of Illness	
Physical disorder	8
Mental disorder ^‡^	8
Multiple health issues	9

^†^ Grandparents and uncle; ^‡^ Mental disorders and addiction-related problems.

## References

[1] Reupert A.E., Cuff R., Drost L., Foste K., van Doesum K.T., Santvoort F.V. (2013). Intervention programs for children whose parents have a mental illness: A review. Med. J. Aust..

[2] Sieh D.S., Visser-Meily J.M.A., Oort F.J., Meijer A.M. (2012). Risk factors for problem behavior in adolescents of parents with a chronic medical condition. Eur. Child Adolesc. Psychiatry.

[3] Leu A., Becker S. (2016). A cross-national and comparative classification of in-country awareness and policy responses to ‘young carers. J. Youth Stud..

[4] Becker F., Becker S. (2008). Young Adult Carers in the UK: Experiences, Needs and Services for Carers Aged 16–24.

[5] Arnett J.J. (2000). Emerging adulthood: A theory of development from the late teens through the twenties. Am. Psychol..

[6] Kraaij V., Garnefski N., de Wilde E.J., Dijkstra A., Gebhardt W., Maes S. (2003). Negative life events and depressive symptoms in late adolescence: Bonding and cognitive coping as vulnerability factors?. J. Youth Adolesc..

[7] Leu A., Frech M., Jung C. (2018). “You don’t look for it”—A study of Swiss professionals’ awareness of young carers and their support needs. Health Soc. Care in Community.

[8] Cass B., Brennan D., Thomson C., Hill T., Purcal C., Hamilton M., Adamson E. (2011). Young Carers: Social Policy Impacts of the Caring Responsibilities of Children and Young Adults.

[9] Moore T., McArthur M. (2007). We’re all in it together: Supporting young carers and their families in Australia. Health Soc. Care Community.

[10] Eley S. (2004). ‘If they don’t recognize it, you’ve got to deal with it yourself’: Gender, young caring and educational support. Gend. Educ..

[11] Gray B., Robinson C., Seddon D. (2009). Invisible children: Young carers of parents with mental health problems—The perspectives of professionals. Child Adolesc. Ment. Health.

[12] Leu A., Frech M., Jung C. (2018). Young carers and young adult carers in Switzerland: Caring roles, ways into care and the meaning of communication. Health Soc. Care Community.

[13] Morrison D.E. (1998). The Search for a Method: Focus Groups and the Development of Mass Communication Research.

[14] Van der Werf H.M., Luttik M.L.A., Francke A.L., Roodbol P.F., Paans W. (2019). Students growing up with a chronically ill family member; a survey on experienced consequences, background characteristics, and risk factors. BMC Public Health.

[15] Schneider S.L. (2013). The international standard classification of education 2011. Class and Stratification Analysis.

[16] Horner S.D. (2000). Using focus group methods with middle school children. Res. Nurs. Health.

[17] Kennedy C., Kools S., Krueger R. (2001). Methodological considerations in children’s focus groups. Nurs. Res..

[18] Roose G.A., John A.M. (2003). A focus group investigation into young children’s understanding of mental health and their views on appropriate services for their age group. Child Care Health Dev..

[19] Lloyd K. (2013). Happiness and well-being of young carers: Extent, nature and correlates of caring among 10 and 11 year old school children. J. Happiness Stud..

[20] Sieh D.S., Meijer A.M., Oort F.J., Visser-Meily J.M.A., Van der Leij D.A.V. (2010). Problem behavior in children of chronically ill parents: A meta-analysis. Clin. Child Fam. Psychol. Rev..

[21] Hennink M., Hutter I., Bailey A. (2010). Qualitative Research Methods.

[22] Metzing-Blau S., Schnepp W. (2008). Young carers in Germany: To live on as normal as possible–a grounded theory study. BMC Nurs..

[23] Hastrup L.H., Van Den Berg B., Gyrd-Hansen D. (2011). Do informal caregivers in mental illness feel more burdened? A comparative study of mental versus somatic illnesses. Scand. J. Public Health.

[24] Ali L., Ahlström B.H., Krevers B., Skärsäter I. (2012). Daily life for young adults who care for a person with mental illness: A qualitative study. J. Psychiatr. Ment. Health Nurs..

[25] Dearden C., Becker S. (2000). Growing up Caring.

[26] Heyman A., Heyman B. (2013). ‘The sooner you can change their life course the better’: The time-framing of risks in relationship to being a young carer. Health Risk Soc..

[27] Dufour M.J., Meijer A.M., Van de Port I., Visser-Meily J.M.A. (2006). Daily hassles and stress in the lives of children with chronically ill parents. Ned. Tijdschr. Voor De Psychol. En Haar Grensgebieden.

[28] Barkmann C., Romer G., Watson M., Schulte-Markwort M. (2007). Parental physical illness as a risk for psychosocial maladjustment in children and adolescents: Epidemiological findings from a national survey in Germany. Psychosomatics.

[29] Pakenham K., Cox S. (2014). The effect of parental illness and other ill family members on the adjustment of children. Ann. Behav. Med..

[30] Östlund U., Persson C. (2014). Examining family responses to family systems nursing interventions: An integrative review. J. Fam. Nurs..

[31] Benzein E., Olin C., Persson C. (2015). ‘You put it all together’–families’ evaluation of participating in Family Health Conversations. Scand. J. Caring Sci..

[32] Benzein E., Johansson P., Årestedt K.F., Saveman B.I. (2008). Nurses’ attitudes about the importance of families in nursing care: A survey of Swedish nurses. J. Fam. Nurs..

[33] Broekema S., Luttik M.L.A., Steggerda G.E., Paans W., Roodbol P.F. (2018). Measuring change in nurses’ perceptions about family nursing competency following a 6-day educational intervention. J. Fam. Nurs..

[34] Sempik J., Becker S. (2013). Young Adult Carers at School: Experiences and Perceptions of Caring and Education.

[35] Accius J. (2018). Breaking stereotypes: Male family caregivers. Innov. Aging.

[36] Mott J., Schmidt B., MacWilliams B. (2019). Male Caregivers: Shifting roles among family caregivers. Clin. J. Oncol. Nurs..

[37] Cancian F.M., Oliker S.J. (2000). Caring and Gender.

